# Women compared with men work harder for small rewards

**DOI:** 10.1038/s41598-023-32391-0

**Published:** 2023-04-04

**Authors:** Carolin A. Lewis, Melina Grahlow, Anne Kühnel, Birgit Derntl, Nils B. Kroemer

**Affiliations:** 1 Department of Psychiatry and Psychotherapy, Tübingen Center for Mental Health, University of Tübingen, Tübingen, Germany; 2 Emotion Neuroimaging Lab, Max Planck Institute for Human Cognitive and Brain Sciences, Leipzig, Germany; 3 International Max Planck Research School on Neuroscience of Communication: Function, Structure, and Plasticity, Leipzig, Germany; 4 Graduate Training Centre of Neuroscience, University of Tübingen, Tübingen, Germany; 5 Department of Psychiatry and Psychotherapy, University of Bonn, Venusberg Campus 1, 53127 Bonn, Germany

**Keywords:** Psychology, Human behaviour

## Abstract

In cost–benefit decision-making, women and men often show different trade-offs. However, surprisingly little is known about sex differences in instrumental tasks, where physical effort is exerted to gain rewards. To this end, we tested 81 individuals (47 women) with an effort allocation task, where participants had to repeatedly press a button to collect food and money tokens. We analyzed the motivational phases of invigoration and effort maintenance with varying reward magnitude, difficulty, and reward type. Whereas women and men did not differ in invigoration, we found that women showed higher effort maintenance as well as higher subjective wanting and exertion ratings for small rewards compared with men. Notably, men increased their effort more than women for higher rewards to match women’s levels of performance. Crucially, we found no sex differences depending on reward type or difficulty, indicating that sex differences were specific to the encoding of the magnitude of benefits, not costs. To summarize, women exerted higher physical effort for small rewards, which corresponded with an elevated subjective value in women compared with men. Therefore, sex differences in perceived reward magnitude may contribute to differential behavioral preferences highlighting the potential of cost–benefit decision-making to provide insights about potential mechanisms.

## Introduction

No bees, no honey—no work, no money. The willingness to expend effort is critical in human behavior. The amount of effort we spend depends on the goals we pursue: we study more to get good grades or exercise harder for a bikini body. Put formally, we determine whether an action is worth pursuing by integrating potential benefits with the cost of an action, which is reflected in a cost–benefit trade-off^1,2^.

Cost–benefit valuations are extensively researched in the decision-making literature (e.g.^3,4^), in particular how decision costs such as delay or uncertainty decrease the subjective value of a reward (i.e., value-based decision-making). So far, it has been shown that women and men differ in important aspects of value-based decision-making (for review, see^5^): For example, men show biases towards maximizing rewards even if this strategy is not optimal, while women seek frequent but smaller rewards. Compared with men, women are more concerned about suboptimal choices in their decision-making strategy^6,7^, and prefer safe options when they lost a reward in a previous decision^8^. Concurrently, men were overall more likely to take risks than women^9^. During reinforcement learning, women outperformed men in learning from positive feedback, while men had enhanced inhibitory control under interference than women^10^. Taken together, women and men show specific preferences to resolve common trade-offs in cost–benefit decision-making that may contribute to differences in reward-related behavior.

Another operationalization of value-based decisions is the allocation of effort, where effort refers to the intensity of mental and/or physical work that individuals apply to obtain some reward^11^. Individuals are considered to exert effort by estimating the expected benefit and the perceived costs to receive a reward^12–14^. The perceived reward value may inform the expected benefit of the effort^15^, which is usually reflected in an effort boost for higher rewards^14^. Sex differences in instrumental physical effort have been reported, with women preferring easy trials with smaller rewards and men preferring difficult trials with higher rewards^16^. However, the nature of this sex-specific behavioral variability in instrumental physical effort is still elusive, e.g., if this sex difference depends on reward magnitude, task difficulty or an interaction of both.

We recently developed and validated a frequency-based version of the effort allocation task (adapted from^13^). Similar to lever pressing in preclinical research^17^, participants collect food and money tokens by repeatedly pressing a button^14^. The task captures two motivational phases: invigoration and effort maintenance. Invigoration describes how quickly a participant ramps up effort; it is associated with subjective wanting and mostly insensitive to effort costs. In contrast, effort maintenance relates to how durably a participant keeps this level of effort^18^. Consequently, effort maintenance is associated with both subjective wanting as well as exertion and it is highly sensitive to the costs of effort. Moreover, we previously reported associations of invigoration and effort maintenance with Carver and White’s^19^ behavioral inhibition system (BIS) and behavioral activation system (BAS), with average effort correlating positively with BIS scores^20^. Taken together, the effort allocation task and its associations with subjective wanting, exertion as well as the BIS/BAS scales provide a good opportunity to elaborate sex differences in instrumental physical effort.

In this study, we aimed at extending previous results of sex differences in value-based decision-making to a task including also physical effort allocation. To this end, we re-analyzed a previously collected data set (^14^, see “Methods”) and tested whether women and men would differ in the motivational phases of invigoration and effort maintenance as measured via the effort allocation task. We predicted invigoration and effort maintenance using reward magnitude (low vs. high), difficulty (easy vs. hard), and reward type (food vs. money) as predictors. We further assessed associations of sex with the subjective ratings of wanting, which relates to the benefits of an action, and exertion, which relates to the costs of an action, as well as sex-specific differences on the BIS/BAS scales. Based on previous results in value-based decision-making tasks, we hypothesized that women and men show differences in both behavior and subjective ratings, and explored if sex differences depend on reward magnitude, task difficulty, or reward type.

## Results

### Women have higher BIS and BAS drive scores than men

We previously reported associations of invigoration and effort maintenance with the BIS/BAS scales in the same sample. We found that average effort correlated positively with BIS scores^20^, but did not examine sex differences. Here, we aimed to describe the sample more precisely for our re-analysis and tested for previously described sex differences on the BIS/BAS scales^21^. Similar to Strobel et al.^21^, women had significantly higher BIS scores than men, *t*(79) = 2.14, *p* = 0.035, but BAS overall scores did not differ between sexes, *t*(79) = 1.66, *p* = 0.101. Women also had significantly higher scores on the subscale BAS Drive than men, *t*(79) = 2.41, *p* = 0.018. The subscales BAS Fun Seeking, *t*(79) = −0.13, *p* = 0.894, and BAS reward responsiveness, *t*(79) = 1.55, *p* = 0.126, did not differ significantly between women and men (Table 1).

**Table 1:** Means (standard deviations) and statistics of behavioral inhibition system (BIS) and behavioral activation system (BAS) scales.

	Mean (SD)		*t*-value	*p*-value
	Female	Male		
BIS	21.28 (3.51)	19.59 (3.48)	2.14	0.035*
BAS	42.17 (4.15)	40.53 (4.72)	1.66	0.101
BAS drive	12.70 (1.85)	11.68 (1.95)	2.41	0.018*
BAS fun seeking	12.38 (1.88)	12.44 (2.00)	−0.13	0.894
BAS reward responsiveness	17.09 (1.77)	16.41 (2.15)	1.55	0.126

*P*-values with an asterisk indicate significance.

As previously reported^20^, average effort correlated positively with BIS scores, *r*_*p*_(79) = 0.29, *p* = 0.009, but not with BAS scores, *r*_*p*_(79) = −0.09, *p* = 0.410. To further examine the sex difference we found for the BIS scores, we ran correlation analyses for average effort and BIS scores separately for women and men. We found that average effort correlated positively with BIS scores in women, *r*_*p*_(45) = 0.29, *p* = 0.045, but not in men, *r*_*p*_(32) = 0.18, *p* = 0.321.

### Women and men differ in effort maintenance, but not invigoration

**Figure 1:**
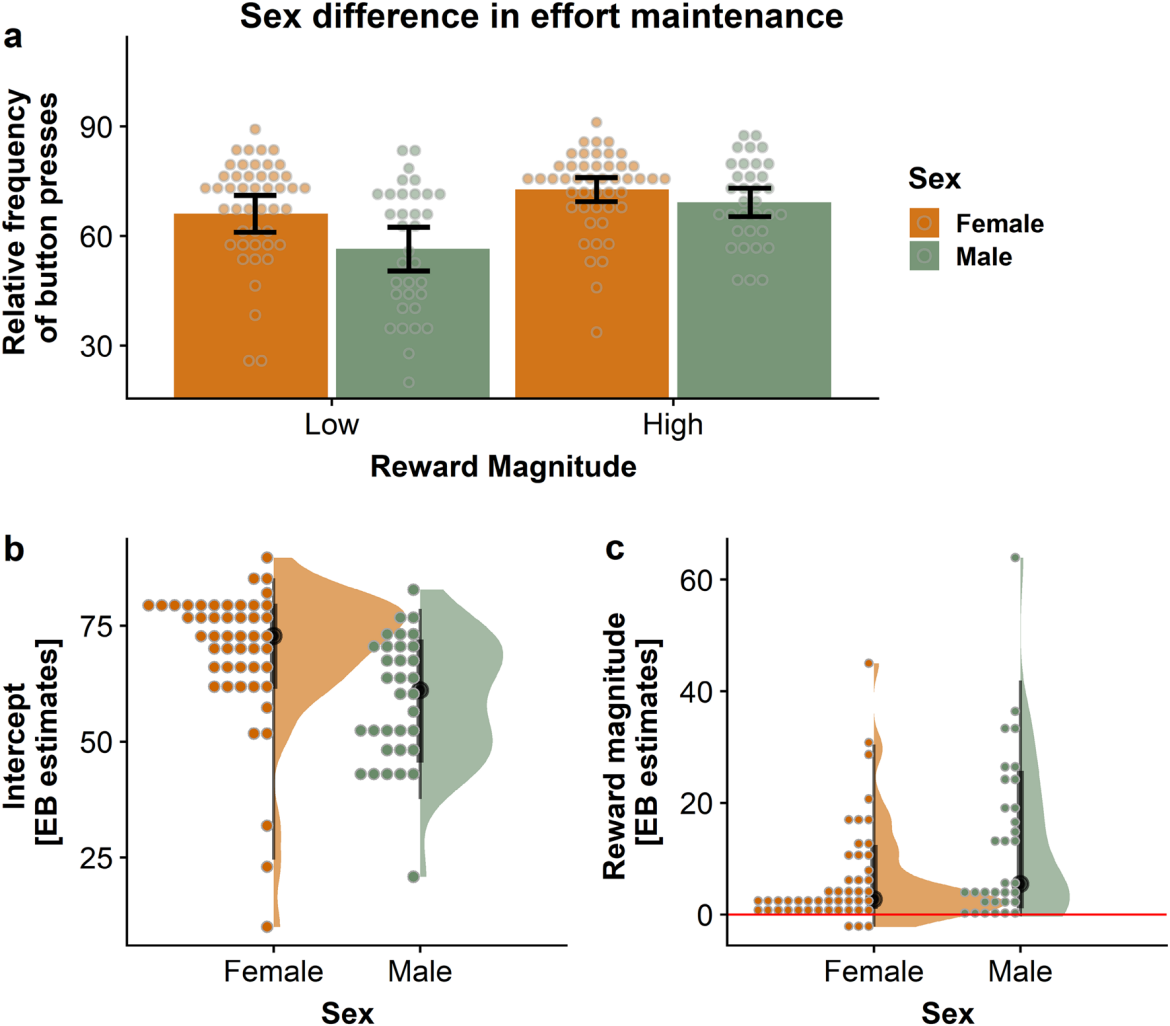
Women and men differ in effort maintenance, depending on reward magnitude. (**a**) Trial-based data showing that women had overall higher effort maintenance than men (main effect of sex, *p* = 0.003). Women generally outperformed men for small rewards, but when more reward was at stake, men adjusted their effort to match women’s performance (interaction sex × reward magnitude, *p* = 0.022). (**b,c**) show empirical Bayes estimates (EB).

To estimate sex differences, we used mixed-effects models predicting either invigoration slopes or effort maintenance (operationalized as average relative frequency of button presses), using the factors reward magnitude (low vs. high), difficulty (easy vs. hard), reward type (food vs. money), and the interaction between reward magnitude × difficulty (Table 2). Women and men did not differ in invigoration, *b* = -0.05, *t*(76) = −0.01, *p* = 0.989. However, we found a main effect of sex for effort maintenance, with women having higher effort maintenance than men for small rewards (or men having lower effort maintenance than women for small rewards), *b* = −9.92, *t*(76) = −3.02, *p* = 0.003. A significant sex × reward magnitude interaction showed that women and men adjusted their performance differentially in response to higher reward magnitudes: men increased their performance in response to higher rewards significantly more than women leading to comparable performance in the high reward condition (i.e., higher slopes for reward magnitude), *b* = 6.01, *t*(76) = 2.33, *p* = 0.022 (Fig. 1). We found no sex differences depending on reward type or difficulty, nor a significant interaction of reward type × difficulty (all *p* > 0.05).

**Table 2:** Estimates of mixed-effects models.

	Coefficient	Standard error	*t*-ratio	*p*-value
Invigoration				
Intercept	55.32	1.71	32.41	< 0.001*
Sex	0.05	3.53	0.01	0.989
Sex × reward magnitude	0.91	2.55	0.36	0.723
Sex × difficulty	−0.40	1.54	−0.26	0.798
Sex × reward type	0.18	1.70	0.11	0.915
Effort maintenance				
Intercept	64.24	1.59	40.43	< 0.001*
Sex	−9.92	3.28	−3.02	0.003*
Sex × reward magnitude	6.01	2.58	2.33	0.022*
Sex × difficulty	−2.35	2.04	−1.15	0.252
Sex × reward type	0.65	1.86	0.35	0.727
Wanting				
Intercept	67.43	2.26	29.90	< 0.001*
Sex	−9.62	3.65	−2.64	0.010*
Reward magnitude	13.41	1.66	8.06	< 0.001*
Sex × reward magnitude	9.31	3.01	3.10	0.003*
Exertion				
Intercept	64.36	2.67	24.09	< 0.001*
Sex	−7.95	4.37	−1.82	0.073
Reward magnitude	11.18	1.87	5.98	< 0.001*
Sex × reward magnitude	10.51	3.47	3.03	0.004*

Variables were coded as follows: sex (male = 0, female = 1), reward magnitude (low = 0, high = 1), difficulty (low = 0, high = 1), reward type (money = 0, food = 1).

*P*-values with an asterisk indicate significance.

In a follow-up analysis, we also examined the total wins of the effort allocation task to see if women or men were overall more successful in earning rewards. Regarding the total points won (i.e., pooled over money and food wins), women were more successful than men, *b* = −3.44, *t*(76) = −2.43, *p* = 0.018.

### Women and men differ in subjective ratings of wanting and exertion

**Figure 2:**
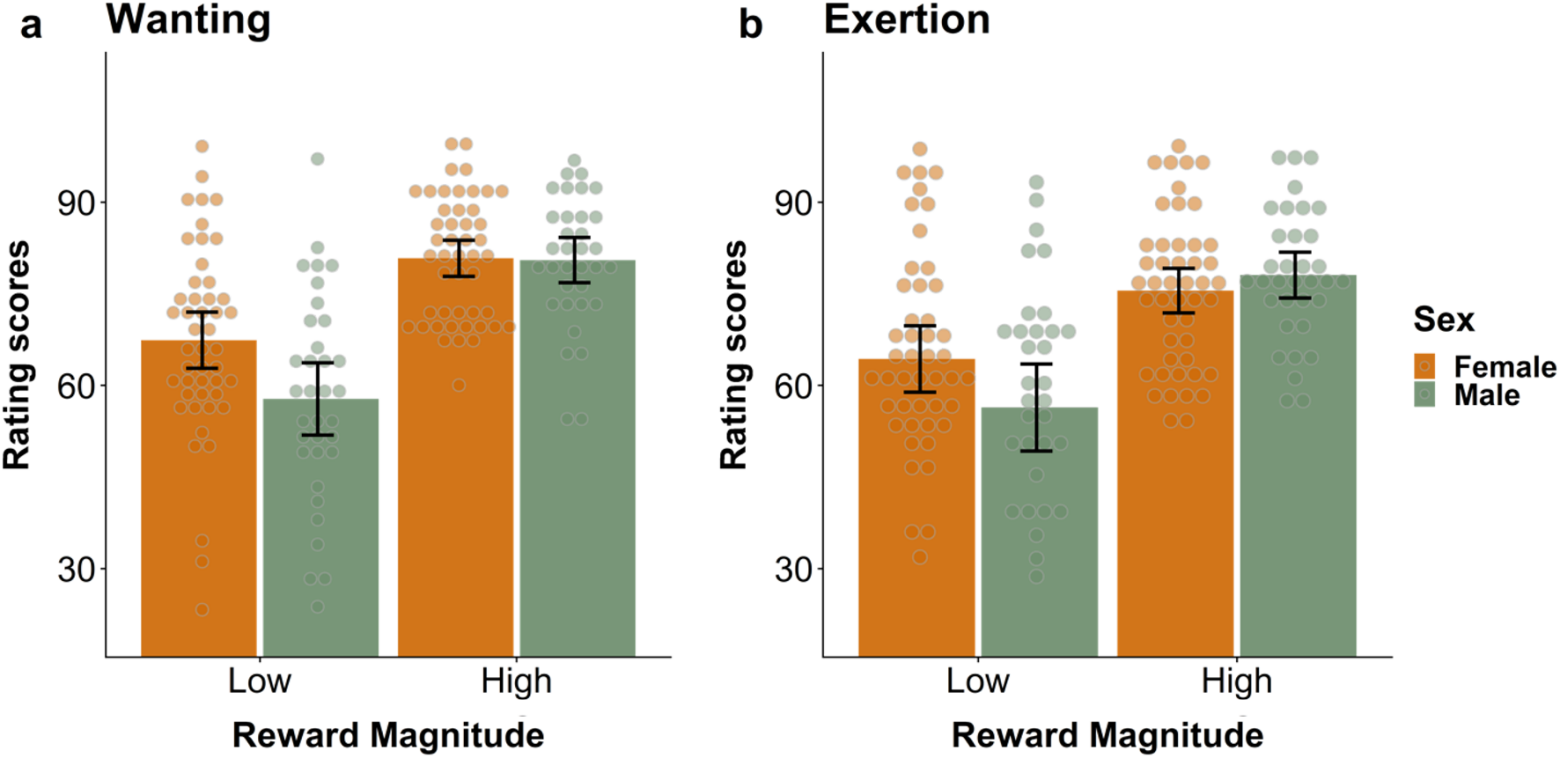
Women and men differed in subjective ratings of wanting and exertion. (**a**) Both women and men had higher wanting ratings for higher rewards than smaller rewards, *p* < 0.001, but women wanted smaller rewards more than men did, *p* = 0.003. (**b**) Both women and men reported to put in more effort for higher rewards than for smaller rewards, *p* < 0.001, and women reported more exertion for smaller rewards than men, *p* = 0.004.

For wanting, we found main effects of sex, with women overall having higher wanting ratings than men, *b* = −9.62, *t*(76) = −2.64, *p* = 0.010, and of reward magnitude, i.e., both women and men wanted higher rewards more than lower rewards, *b* = 13.41, *t*(76) = 8.06, *p* < 0.001. The interaction of Sex x Reward Magnitude was also significant, meaning that women had higher wanting ratings than men for smaller rewards, *b* = 9.31, *t*(76) = 3.10, *p* = 0.003 (Fig. 2a). For exertion, the main effect of sex was not significant, *b* = −7.95, *t*(76) = −1.82, *p* = 0.073, only the main effect of reward magnitude, i.e., both women and men reported to put in more effort for higher rewards, *b* = 11.18, *t*(76) = 5.98, *p* < 0.001. Similar to wanting, we found a significant interaction of sex × reward magnitude for exertion, with women putting in more effort for smaller rewards than men, *b* = 10.51, *t*(76) = 3.03, *p* = 0.004 (Fig. 2b).

## Discussion

Women and men have specific preferences to resolve common trade-offs in cost–benefit decision-making. However, sex differences in instrumental physical effort are less well understood, especially if sex-specific behavioral variability depends on key factors of the tasks, such as reward magnitude, difficulty, and reward type. To this end, we investigated sex differences in instrumental physical effort in humans using an effort allocation task, which captured the motivational phases invigoration and effort maintenance. Although women and men showed comparable invigoration, women showed overall higher effort maintenance compared with men. More specifically, women outperformed men for small rewards. However, men increased their effort more than women for higher rewards. Notably, women and men showed no behavioral differences when different reward types were at stake or greater difficulty was required to obtain a reward, indicating that sex differences were specific to the encoding of potential benefits, not costs. This interpretation was substantiated by differences in subjective ratings of wanting and exertion because women wanted smaller rewards more and reported higher exertion compared with men, whereas ratings were comparable for large rewards. To summarize, we found sex differences in instrumental physical effort expenditure, which became evident in both objective and subjective measures. By showing that sex-specific behavioral variability depended on reward magnitude, and not on reward type or task difficulty, we contribute to an improved understanding of sex differences in instrumental physical effort that may facilitate differential preferences.

Our results showed that women and men differed in instrumental physical effort, depending on reward magnitude. This difference was mainly driven by the fact that women put in more effort for smaller rewards, while men worked about as hard as women when larger rewards were at stake. We thereby extended results from studies on sex differences in value-based decision-making, e.g., where women seek certain, smaller rewards, while men preferred larger, but less consistent rewards^7^. Effort-based versions of cost–benefit paradigms, like the effort allocation task used in our study, focus on the costs of physical effort to obtain rewards. Here, the perceived reward value is considered to inform the expected benefit of the effort^15^, which usually leads to higher effort for larger rewards^14^. Our results show that women and men evaluated the perceived reward value differently and, thus, allocated their effort differently in light of small vs. large rewards. When more reward was at stake, men increased their effort more than women to match their performance. Consequently, men were more opportunistic, while women also worked more for smaller rewards. In turn, women ascribed higher value to small rewards than men, which was also corroborated by women’s higher subjective wanting and exertion ratings for smaller rewards compared with men.

Of note, invigoration refers to automatic processes related to motivational drive, while effort maintenance rather describes an active decision of allocating physical effort^11,22^. Moreover, effort maintenance refers to how much effort one is *willing* to spend to gain rewards, rather than how much effort one is (physically) *able* to exert. Since we did not find a sex difference in invigoration, but only in effort maintenance, we can assume that women actively chose to put in effort for both small and high rewards. Women weighed the benefits of smaller rewards higher than men, but women may have also valued effort itself higher than men. Effort can add substantial value to both rewards and to effort itself (‘The Effort Paradox’^11,23^). We can not rule out that women in our study might have valued the rewarding experience of exerting effort higher than men, which boosted the valuation of smaller rewards. Regarding the total points won, the female strategy of putting in effort also for small rewards can be seen as more successful than the male strategy of presumably saving effort costs for small rewards. Another line of argument was the possibility that women and men allocate physical effort differently depending on task demands, such as difficulty, e.g., Treadway et al.^16^ found that women preferred easy trials with smaller rewards and men preferred difficult trials with higher rewards. However, we did not find a sex difference in effort maintenance depending on difficulty to obtain a reward. Our results were further corroborated by experiences from previous studies, in which behavioral differences in effort allocation became evident in the face of small rewards, whereas for larger rewards, most individuals give their very best (e.g.^15^). Taken together, our results suggest that sex differences in instrumental physical effort depend on reward magnitude, with women weighing the benefits of smaller rewards higher than men.

Moreover, we found that women had significantly higher BIS and BAS Drive scores than men. Higher BIS scores in women have repeatedly been shown in validation samples (e.g.^19,21^). BIS stands for the motivation to avoid aversive outcomes, and, thus, women in our study felt ‘worried when they thought they have done poorly at something’, more than men. Also, compared with men, women in our study ‘went out of their way to get things they wanted’, as described by higher BAS Drive scores (motivation to pursuit desired goals). The sex difference in both behavioral inhibition and activation may thus contribute to the finding that women in our study generally put in more effort for rewards than men. In the same sample, we previously reported a positive correlation of average effort with BIS scores^20^, and extended this result here by showing that this correlation seemed to be mainly driven by women. Consequently, higher BIS scores in women in our analysis may further explain why women had overall higher effort maintenance than men: women avoided an aversive situation, i.e., ‘doing poorly at something’, by ramping up their effort to fulfill the task requirements. This fits well with the finding that effort can have signaling functions in social settings, as it is easily detected by self and others: by putting in more effort, women may express more commitment and dedication to the task^24,25^. Men, with lower BIS scores, might have been less affected by this, and, thus, had a rather opportunistic motivation in performing the task.

Furthermore, personality factors and gender roles need to be considered when discussing the present findings. In large community samples, women have been found to be more worried (Neuroticism), social (Extraversion), inquisitive (Openness), caring (Agreeableness), and responsible (Conscientiousness) than men (e.g.^26^). Especially the female personality traits neuroticism and conscientiousness can be hypothesized to make them more prone to be attentive and diligent when solving tasks. These self-concepts can influence behavior and are thought to be influenced by experience, social desirability concerns, and societal norms^27^. According to social structural theories, gender differences in behavior stem from shared social expectations of how women and men should behave^28^. These gender roles are internalized very early in life and both shape personality traits and behavior. Thus, women and men seek different experiences to maximize their outcomes^29^. Regarding the results of our study, women and men chose different strategies to maximize their reward gains: women put in more effort than men for small rewards, whereas men increased their effort more for higher rewards so that the performance for high rewards matches the performance of women. For women, success (or an optimal performance in the task) presumably also included to perform well on the task, which also comprised to follow the task instructions thoroughly. In other words, for women it was socially desirable to solve the task conscientiously and to give their very best for both low and high reward trials. For men, a competitive task solving strategy was socially more desirable, i.e., behavior which focused on overall maximizing reward gain. In this vein, men might have saved physical energy on low reward trials to then maximize their effort for high reward gains. A well-established sex/gender difference for the Iowa Gambling Task supports this notion, showing that men focus on long-term pay-off more than women^30^. If and how personality factors and gender roles also influence instrumental physical effort as measured here cannot fully be addressed by the results of this study, but should be investigated in future research.

The present study has several limitations which could guide future research. First, we did not measure sex hormone levels, e.g., estradiol, progesterone, or testosterone. Sex hormone receptors are densely present along midbrain areas and thereby modulate decision-making processes by interacting with relevant neurotransmitter systems (for review, see^5,31^). It remains an open question if and how sex hormones also influence physical effort expenditure and thereby contribute to sex differences. Second, we did not assess gender-related attributes and merely split the sample into biological females and males. However, we can not rule out if self-perceived feminine or masculine traits may also contribute to behavioral differences between women and men, i.e., if individuals allocate physical effort differently if they consider themselves as being for example more risk-taking (typically male) or more conscientious (typically female).

Value-based decision-making shows sex differences in the integration of benefits and costs, but potential biases in the allocation of physical effort when rewards are at stake were largely elusive. We investigated sex differences in instrumental physical effort and found that women showed overall higher effort maintenance than men. More specifically, women had higher effort maintenance than men for small rewards, while for higher rewards, men adjusted their effort to match women’s performance. In line with behavioral differences, women also reported higher wanting and exertion for smaller rewards compared with men. Taken together, our results highlight sex differences in instrumental physical effort and subjective wanting and exertion that are explained by an elevated subjective value of small rewards in women compared with men. Since these sex differences were not specific to task difficulty or reward types, we conclude that sex differences in instrumental physical effort depended on the encoding of potential benefits, not costs. We thereby contribute to the understanding of sex-specific behavioral variability on motivated behaviors and underline the potential of cost–benefit decision-making to understand potential mechanisms in several domains, such as education and mental health.

## Methods

### Participants

85 individuals participated in the study and completed two sessions each: one session took place during stimulation of the cymba conchae (taVNS) and the other one during sham stimulation at the earlobe. Methods and results of the taVNS stimulation are reported elsewhere^14,32^ and are thus not further reported in this manuscript. The total sample size for the current analysis was N = 81 after exclusion of four participants (n = 3: did not finish the second experimental session, for example due to sick leave, n = 1: was assigned an incorrect maximum of button press frequency precluding comparison of the two sessions). Half of the participants completed the effort task during left-sided taVNS and the other half completed the effort task during right-sided taVNS. As determined by a telephone interview, participants were physically and mentally healthy, German speaking, and right-handed (47 women: *M*_*age*_ = 24 ± 3 years, *M*_*BMI*_ = 22.4 ± 2.9 kg/m^2^; 34 men: *M*_*age*_ = 25 ± 4 years, *M*_*BMI*_ = 24.0 ± 3.0 kg/m^2^). The study was approved by the local ethics committee (the institutional review board of the Faculty of Medicine, University of Tübingen) and was conducted in accordance with the ethical code of the World Medical Association (Declaration of Helsinki). Participants took part voluntarily and provided written informed consent at the beginning of Session 1. They received either monetary compensation (32€ fixed amount) or course credit for their participation. Moreover, depending on their task performance, participants received money and a breakfast (cereal + chocolate bar).

### Experimental procedure

The study was designed so that experimental sessions were conducted in a randomized, single-blind crossover fashion. Experimental session started between 7:00 am and 10:15 am and lasted about 2.5 h for each session. Participants were asked to fast overnight (> 8 h prior to the visit). In the beginning of the first session, participants selected their preferred type of cereal out of four options (dried fruits, chocolate, cookies, or honey nut; Peter Kölln GmbH & Co. KGaA, Elmshorn, Germany). It was explained that participants would collect energy and money points depending on their performance in the effort allocation task. The participant’s breakfast serving would consist of cereal and milk scaled according to the energy points earned during the task. During the session, participants could drink water ad libitum.

First, participants completed a set of state ratings^32^ followed by practice trials of the effort allocation task to estimate the maximum frequency of button presses for every individual. A blue ball depicted within a tube appeared on the screen for two initial trials of 10 s length each. By repeatedly pressing a button on the Xbox 360 controller (Microsoft Corporation, Redmond, WA) with their right (dominant) index finger, participants could move the ball upwards within the tube. A blue tangent line on the vertical axis was also moved by moving the ball upwards, marking the highest position reached by the ball so far. This line would depict the maximum frequency of button presses achieved so far (“peak”) even when participants stopped pressing the button and remained at the highest position, in contrast to the ball. Participants were encouraged to push the line as high as they could. Next, participants completed a short practice analogous to the effort task consisting of eight trials that comprised all possible combinations of reward magnitude (low vs. high), difficulty (easy vs. hard), and reward type (food vs. money) presented in a randomized order including a short break after half of the trials. By use of these practice trials, the maximum frequency of button presses was updated if participants exceeded the previous level achieved during training. After completing the practice trials, participants received feedback about the reward they would have won as a reference for the following experiment (for details, see^14^).

After the tasks, participants received their breakfast and a snack according to the food reward (“energy”) points earned. At the end of the first session, participants received their monetary wins as part of the compensation. Both sessions took place within a week (usually within 3–4 days), were conducted at approximately the same time, and followed the same standardized protocol. Participants either received monetary compensation (32€ fixed amount + wins of Session 2) or course credit (+ wins of Session 2) after the second session.

### Effort allocation task

By exerting effort (i.e., repeatedly pressing a button with the right index finger), participants collected food and money tokens throughout the effort allocation task. Analogous to preclinical studies of lever pressing^17^, the task used frequency of button presses instead of grip force to measure physical effort (adapted from^13^). Tokens were exchanged for calories (cereal + chocolate bar as snack) or money at a rate of 1 kcal or 1 cent per five tokens at the end of the session.

**Figure 3:**
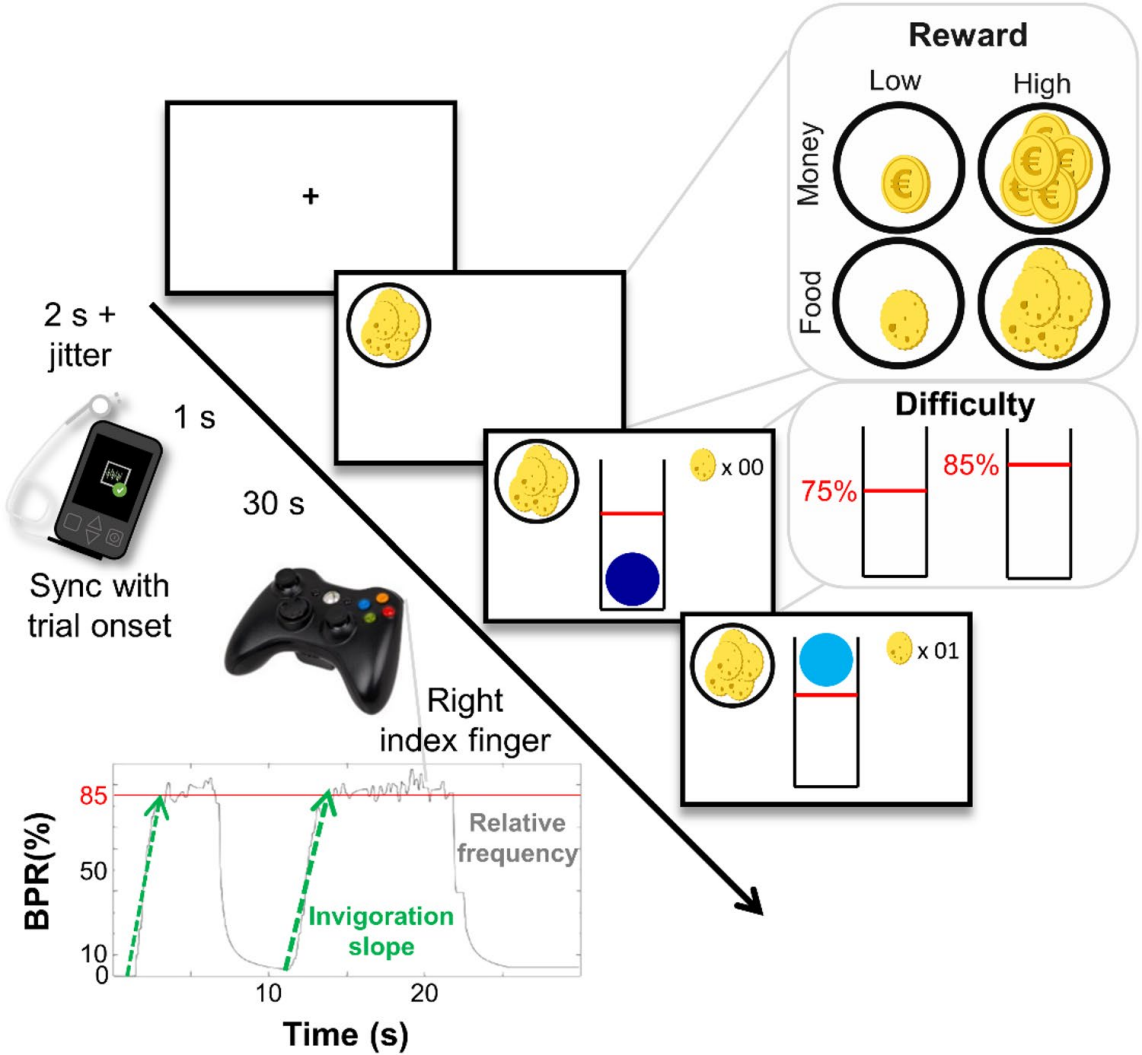
Schematic depiction of the effort allocation task. First, fixation cross is shown, followed by the reward cue. To earn reward, participants have to keep a ball above the red line by repeatedly pressing a button with their right index finger. Reward magnitude (low vs. high), difficulty (easy vs. hard), and reward type (food vs. money) were manipulated as task conditions. The lower left graph shows a representative time series of a high-difficulty trial, depicting effort output as button press rate, BPR, in % relative to the maximum frequency of the participant. Invigoration slopes captured how quickly participants reach effortful behavior during a trial to collect the reward. Effort maintenance relates to the average relative frequency on the trial. Figure taken from^14^ under CC BY license (https://creativecommons.org/licenses/by/4.0/); no changes have been made to the figure.

A prospective reward, which could be either food (indicated by a cookie) or money (indicated by a coin), was presented for 1 s at the start of every trial. The magnitude of the reward at stake was varied as one symbol signaled a low magnitude (1 point/s) whereas several symbols indicated a high reward magnitude (10 points/s). Participants won 362.8 kcal and €3.78 per session on average. Following, a blue ball contained within a tube was presented on the screen. Participants were instructed to vertically move the ball above a certain difficulty level by repeatedly pressing a button on the controller with the right index finger to earn reward points. Difficulty corresponded to a relative frequency threshold and was indicated by a red line. Reward points were accumulated and tracked by a counter in the upper right corner of the screen (Fig. 3) for every second that the ball was kept above the threshold (indicated by a change of color from dark to light blue). By alternating the red threshold line between 75 and 85% (counterbalanced order across participants) of the individual maximum frequency, difficulty was varied. We used a moving average algorithm with exponential weighting (λ = 0.6) to smooth the movement of the ball for display on screen. Hence, the ball fell quickly yet slowed down when participants stopped working or reduced the frequency.

Participants were presented sequentially with two visual analogue scales inquiring about exertion and wanting of the reward at stake after every effort phase of each of the 48 trials comprised in the task. Participants were encouraged to take breaks at their convenience to recover during trials, so that they could try to exceed the threshold again, as the task was too difficult to always keep the ball above the red line, as was emphasized in the instructions. Participants could take a short break to recuperate after completing the first half of the task. The total amount of tokens they had collected was shown on the screen after completing the task. Only completed sessions were rewarded in tokens. The task was presented using Psychophysics toolbox v3^33^ in MATLAB v2017a.

### BIS/BAS scale

We used the German version of the BIS/BAS scale^21^, originally developed by Carver and White^19^. The BIS/BAS scale measures two motivational systems: the behavioral inhibition system (BIS), which corresponds to motivation to avoid aversive outcomes, and the behavioral activation system (BAS), which describes motivation to approach goal-oriented outcomes. The questionnaire has 24 items with 4-point Likert scale responses (from 1 = ‘very true for me’, to 4 = ‘very false for me’). One of the four subscales correspond to the BIS and comprises items like ‘I worry about making mistakes’ or ‘I feel worried when I think I have done poorly at something’. The three components of BAS compose the remaining three subscales. BAS Drive measures the motivation to pursuit desired goals, e.g., ‘I go out of my way to get things I want’. BAS Reward Responsiveness focuses on positive responses to pleasant reinforcers, e.g., ‘When I’m doing well at something, I love to keep at it’. BAS Fun Seeking comprises items that measure the motivation to approach new rewards spontaneously, e.g., ‘I crave excitement and new sensations’. We previously reported associations of invigoration and effort maintenance with BIS/BAS in the same sample, but did not examine sex differences^20^.

### Data analysis

To isolate the facets invigoration and effort maintenance, we divided the behavioral data into work and rest segments (see also^14^). Invigoration was estimated with the slope of the transition between relative frequency of button presses during a rest segment and their initial plateau during the following work segment (MATLAB findpeaks function). Effort maintenance was the average frequency of button presses during a trial capturing how much effort participants produce over time.

Invigoration and effort maintenance estimates at the trial level were then entered in a mixed-effects analysis as implemented in hierarchical linear models (HLM^34^). We used two univariate mixed-effects models, as both outcomes were only moderately correlated, *r* = 0.286, 95% CI [0.25, 0.32]. We predicted either invigoration or effort maintenance using the following predictors: stimulation (sham vs. taVNS), reward magnitude (low vs. high), difficulty (easy vs. hard), reward type (food vs. money, all dummy coded), the interaction between reward magnitude × difficulty, as well as interactions of stimulation with all these terms. At the participant level, we included stimulation order, stimulation side (both mean centered), BMI, and sex. Intercepts and slopes were modeled as random effects to account for individual deviations from fixed group effects. As detailed in^14^, the taVNS stimulation effect was accounted for by including stimulation condition (taVNS vs. sham) together with all interactions of stimulation with the other predictors of the model. The order of stimulation conditions and stimulation side were controlled for at the participant level and results of the taVNS stimulation were already reported elsewhere^14^. We found no sex-specific effects of taVNS vs. sham, stimulation order, or stimulation side, and thus pooled both sessions in our current analysis.

Moreover, to assess specific associations of sex with the subjective ratings of wanting (related to benefits of action) and exertion (related to costs of action), we used mixed-effects models as implemented in R (lmerTest), predicting wanting or exertion as outcomes, respectively, and using sex and reward magnitude as predictors.

### Statistical threshold and software

We used a two-tailed α ≤ 0.05 for the analyses of our main research question: Do women and men differ in invigoration or effort maintenance? Mixed-effects analyses were conducted with HLM v7^35^ and lmerTest in R^36^. To determine the evidence for the alternative hypothesis provided by our results, we calculated corresponding BFs based on individual empirical Bayes estimates. Effort data was processed with MATLAB vR2017-2019a and SPSS v24. Results were plotted with R v4.1.0 (R Core Team, 2017).

## Data Availability

Source data are provided with a previous publication^14^. Trial-based behavioral data that was used to conduct all analyses are publicly available on OSF: https://osf.io/58r3c/?view_only=5d1ccee7d67b464bb6f40ebe7ebc844b.

## References

[1] Westbrook A, Braver TS (2015). Cognitive effort: A neuroeconomic approach. Cogn. Affect. Behav. Neurosci..

[2] Phillips PE, Walton ME, Jhou TC (2007). Calculating utility: Preclinical evidence for cost-benefit analysis by mesolimbic dopamine. Psychopharmacology.

[3] Rangel A, Camerer C, Montague PR (2008). A framework for studying the neurobiology of value-based decision making. Nat. Rev. Neurosci..

[4] Zald DH, Treadway MT (2017). Reward processing, neuroeconomics, and psychopathology. Annu. Rev. Clin. Psychol..

[5] Ambrase A, Lewis CA, Barth C, Derntl B (2021). Influence of ovarian hormones on value-based decision-making systems: Contribution to sexual dimorphisms in mental disorders. Front. Neuroendocrinol..

[6] Byrne KA, Worthy DA (2015). Gender differences in reward sensitivity and information processing during decision-making. J. Risk Uncertain..

[7] Cornwall AC, Byrne KA, Worthy DA (2018). Gender differences in preference for reward frequency versus reward magnitude in decision-making under uncertainty. Pers. Individ. Differ..

[8] Lee TMC, Chan CCH, Leung AWS, Fox PT, Gao JH (2009). Sex-related differences in neural activity during risk taking: An fMRI study. Cereb. Cortex.

[9] Byrnes JP, Miller DC, Schafer WD (1999). Gender differences in risk taking: A meta-analysis. Psychol. Bull..

[10] Evans KL, Hampson E (2015). Sex-dependent effects on tasks assessing reinforcement learning and interference inhibition. Front. Psychol..

[11] Inzlicht M, Shenhav A, Olivola CY (2018). The effort paradox: Effort is both costly and valued. Trends Cogn. Sci..

[12] Meyniel F, Safra L, Pessiglione M (2014). How the brain decides when to work and when to rest: Dissociation of implicit-reactive from explicit-predictive computational processes. Plos Computat. Biol..

[13] Meyniel F, Sergent C, Rigoux L, Daunizeau J, Pessiglione M (2013). Neurocomputational account of how the human brain decides when to have a break. Proc. Natl. Acad. Sci. USA.

[14] Neuser MP (2020). Vagus nerve stimulation boosts the drive to work for rewards. Nat. Commun..

[15] Kroemer NB (2014). Balancing reward and work: Anticipatory brain activation in NAcc and VTA predict effort differentially. Neuroimage.

[16] Treadway MT, Buckholtz JW, Schwartzman AN, Lambert WE, Zald DH (2009). Worth the 'EEfRT'? The effort expenditure for rewards task as an objective measure of motivation and anhedonia. PLoS ONE.

[17] Salamone JD, Yohn SE, Lopez-Cruz L, San Miguel N, Correa M (2016). Activational and effort-related aspects of motivation: Neural mechanisms and implications for psychopathology. Brain.

[18] Kroemer NB, Burrasch C, Hellrung L (2016). To work or not to work: Neural representation of cost and benefit of instrumental action. Prog. Brain Res..

[19] Carver CS, White TL (1994). Behavioral inhibition, behavioral activation, and affective responses to impending reward and punishment: the BIS/BAS scales. J Pers. Soc. Psychol..

[20] van den Hoek Ostende MM, Neuser MP, Teckentrup V, Svaldi J, Kroemer NB (2021). Can't decide how much to EAT? Effort variability for reward is associated with cognitive restraint. Appetite.

[21] Strobel A, Beauducel A, Debener S, Brocke B (2001). Eine deutschsprachige version des BIS/BAS-Fragebogens von Carver und White [A German version of Carver and White's BIS/BAS scales]. Z. Differ. Diagn. Psychol.

[22] Kahneman, D. *Attention and Effort*. Vol. 1063. 218–226 (Prentice Hall, 1973).

[23] Inzlicht M, Campbell AV (2022). Effort feels meaningful. Trends Cogn. Sci..

[24] Bigman YE, Tamir M (2016). The road to heaven is paved with effort: Perceived effort amplifies moral judgment. J. Exp. Psychol. Gen..

[25] Dik G, Aarts H (2007). Behavioral cues to others’ motivation and goal pursuits: The perception of effort facilitates goal inference and contagion. J. Exp. Soc. Psychol..

[26] MacGiolla E, Kajonus PJ (2019). Sex differences in personality are larger in gender equal countries: Replicating and extending a surprising finding. Int. J. Psychol..

[27] Greenwald AG, Poehlman TA, Uhlmann E, Banaji MR (2009). Understanding and using the implicit association test: III. Meta-analysis of predictive validity. J. Pers. Soc. Psychol..

[28] Eagly AH (1987). Sex Differences in Social Behavior: A Social-Role Interpretation.

[29] Eagly AH, Wood W (1999). The origins of sex differences in human behavior: Evolved dispositions versus social roles. Am. Psychol..

[30] van den Bos R, Homberg J, de Visser L (2013). A critical review of sex differences in decision-making tasks: Focus on the Iowa gambling task. Behav. Brain Res..

[31] Barth C, Villringer A, Sacher J (2015). Sex hormones affect neurotransmitters and shape the adult female brain during hormonal transition periods. Front. Neurosci..

[32] Ferstl M (2021). Non-invasive vagus nerve stimulation boosts mood recovery after effort exertion. Psychol. Med..

[33] Brainard DH (1997). The psychophysics toolbox. Spat. Vis..

[34] Raudenbush SW, Bryk AS (2002). Hierarchical Linear Models: Applications and Data Analysis Methods.

[35] Raudenbush SW, Bryk AS, Cheong YF, Congdon RT, Du Toit M (2011). HLM 7.

[36] Kuznetsova A, Brockhoff PB, Christensen RHB (2017). lmerTest package: Tests in linear mixed effect models. J. Stat. Softw..

